# Electrical impedance tomography to measure lung ventilation distribution in healthy horses and horses with left‐sided cardiac volume overload

**DOI:** 10.1111/jvim.16227

**Published:** 2021-08-04

**Authors:** Muriel Sacks, David P. Byrne, Nicolas Herteman, Cristy Secombe, Andy Adler, Giselle Hosgood, Anthea L. Raisis, Martina Mosing

**Affiliations:** ^1^ School of Veterinary Medicine Murdoch University Perth Australia; ^2^ Equine Clinic, Department for Equine Medicine Vetsuisse Faculty, University of Zurich Zürich Switzerland; ^3^ Systems and Computer Engineering Carleton University Ottawa Canada

**Keywords:** center of ventilation, congestive heart failure, EIT, furosemide, pulmonary edema, pulmonary hypertension

## Abstract

**Background:**

Left‐sided cardiac volume overload (LCVO) can cause fluid accumulation in lung tissue changing the distribution of ventilation, which can be evaluated by electrical impedance tomography (EIT).

**Objectives:**

To describe and compare EIT variables in horses with naturally occurring compensated and decompensated LCVO and compare them to a healthy cohort.

**Animals:**

Fourteen adult horses, including university teaching horses and clinical cases (healthy: 8; LCVO: 4 compensated, 2 decompensated).

**Methods:**

In this prospective cohort study, EIT was used in standing, unsedated horses and analyzed for conventional variables, ventilated right (VA*R*) and left (VA*L*) lung area, linear‐plane distribution variables (avg‐max VΔZ_Line_, VΔZ_Line_), global peak flows, inhomogeneity factor, and estimated tidal volume. Horses with decompensated LCVO were assessed before and after administration of furosemide. Variables for healthy and LCVO‐affected horses were compared using a Mann‐Whitney test or unpaired *t*‐test and observations from compensated and decompensated horses are reported.

**Results:**

Compared to the healthy horses, the LCVO cohort had significantly less VA*L* (mean difference 3.02; 95% confidence interval .77‐5.2; *P* = .02), more VA*R* (−1.13; −2.18 to −.08; *P* = .04), smaller avg‐max VΔZ*L*_Line_ (2.54; 1.07‐4.00; *P* = .003) and VΔZ*L*_Line_ (median difference 5.40; 1.71‐9.09; *P* = .01). Observation of EIT alterations were reflected by clinical signs in horses with decompensated LCVO and after administration of furosemide.

**Conclusions and Clinical Importance:**

EIT measurements of ventilation distribution showed less ventilation in the left lung of horses with LCVO and might be useful as an objective assessment of the ventilation effects of cardiogenic pulmonary disease in horses.

**Abbreviations**
bpm: beats per minute
brpm: breaths per minute
CoV_VD_ and CoV_RL_
: ventral to dorsal and right to left center of ventilation
DSS: dependent silent space
EIT: electrical impedance tomography
FIO_2_
: fraction of inspired oxygen
GREIT: Graz consensus reconstruction algorithm for EIT
H1_PE_ & H2_PE_
: horse with pulmonary edema 1&2
ICS: intercostal space
LCVO: left‐sided cardiac volume overload
MUSVM: Murdoch University School of Veterinary Medicine
NSS: nondependent silent space
P_A‐a_O_2_
: alveolar‐to‐arterial oxygen partial pressure difference
PaO_2_
: partial pressure of arterial oxygen
PaCO_2_
: partial pressure of arterial carbon dioxide
PIF_EIT_
: global peak inspiratory expiratory
PEF_EIT_
: global peak inspiratory expiratory
PE: pulmonary edema
ROI: region of interest
VΔZ: ventilation‐induced impedance change
VΔZ: EIT derived tidal variation
VΔZ_*V*_
: regional ventral tidal variation
VΔZc_*V*_
: regional central ventral tidal variation
VΔZ_*CD*_
: regional central dorsal tidal variation
VΔZ_*D*_
: regional dorsal tidal variation
VΔZ*R*_Line_
: number of lines with detected impedance change for the right lung field
VΔZ*L*_Line_
: number of lines with detected impedance change for the left lung field
avg‐max VΔZ*R*_Line_
: average maximum impedance change of all horizontal matrix lines in the right lung
avg‐max VΔZ*L*_Line_
: average maximum impedance change of all horizontal matrix lines in the left lung
VA*R*
: ventilated area of the right lung
VA*L*
: ventilated area of the left lung
VGI: global inhomogeneity index

## 1 INTRODUCTION

Left‐sided cardiac volume overload (LCVO) causes an increase in pulmonary venous pressure and can lead to fluid accumulation in the interstitial lung tissue and alveolar space in horses.^1^ Chronic high intravascular pressure might cause pulmonary vascular remodeling and lead to perivascular inflammation resulting in tissue fibrosis and changes in tissue elasticity as shown in humans.^2^ As such, LCVO can impair the respiratory physiology in horses and, when severe, lead to alveolar pulmonary edema (PE) with risk of collapse or sudden death.^3^ Stall‐side diagnosis of pulmonary disease is often difficult in horses and primarily consists of detection of cough and tachypnoea and the auscultation of loud bronchovesicular sounds or crackles over the thorax.^1^, ^4^ Thoracic radiography and ultrasound often lack sensitivity in horses especially for detection of subclinical and deeper parenchymal pulmonary changes, whereas techniques such as computed tomography lack practicality.^5^

Electrical impedance tomography (EIT) is a noninvasive radiation‐free functional imaging modality for continuous monitoring of pulmonary ventilation.^6^ An electrode belt is placed around the thorax of the horse generating alternating currents from sequential pairs of electrodes that then circulate around the thorax. The intrathoracic tissue allows the passage of current with little impedance; however, the inhalation of gas increases intrathoracic resistance. Resulting impedance change is detected by the remaining electrodes, which form a relative image with respect to the reference baseline of the lungs at the start of inspiration.^7^, ^8^ This modality has been used to evaluate distribution of ventilation, changes in gas flow in the airways and tidal volume in awake and anesthetized horses.^9^, ^10^, ^11^, ^12^, ^13^ Furthermore, noncardiogenic and cardiogenic PE has been assessed with EIT in people, pigs and dogs.^14^, ^15^, ^16^, ^17^, ^18^, ^19^ Changes in EIT variables in response to reduction of extravascular lung water in people demonstrated its potential utility in the clinical management of alveolar PE and pleural effusion in this setting.^20^, ^21^

The aim of this prospective cohort study was to report EIT variables describing ventilation in standing unsedated horses with LCVO and to compare them to a healthy cohort. For the purpose of this study, LCVO was considered present when there was eccentric hypertrophy of either left atrium or ventricle, which is expected to cause secondary pulmonary venous hypertension. We hypothesized that EIT derived data reflecting ventilation will be different between cohorts. Furthermore, we hypothesized that EIT measurements in decompensated LCVO with clinical signs of alveolar PE will be different to those in compensated LCVO with assumed interstitial accumulation of extravascular lung water and tissue fibrosis.

## 2 MATERIALS AND METHODS

### 2.1 Animals

This prospective observational cohort study comprised examination of a total of 14 horses; 12 horses from the Murdoch University, School of Veterinary Medicine, teaching herd (Animal Ethics Committee permit number R3205/19) and 2 horses with clinical signs of decompensated LCVO, as defined below. These 2 horses were clinical cases hospitalized at the Murdoch University School of Veterinary Medicine and the University of Zurich Equine Hospital. Prior to the study, the 12 teaching horses were housed in an irrigated paddock with free access to water and received supplemental feed of roughage.

### 2.2 Healthy cohort

Eight horses from the teaching herd were included in this cohort. Based on long term history, thorough clinical examination and auscultatory findings all horses were deemed free of overt cardiopulmonary disease. Intermittent informal echocardiography and thoracic ultrasonography, performed as part of standard teaching activities, revealed no evidence of subclinical disease. All body condition scores ranged from 4‐6/9.

### 2.3 Cohort with LCVO

Six horses with LCVO of various severities were included in this cohort.

#### 2.3.1 Horses with compensated LCVO

Four horses from the same teaching herd with previously diagnosed compensated heart disease causing LCVO as reflected by left atrial or ventricular enlargement, but without clinical signs at rest were recruited to this cohort. Horses had concurrent aortic and tricuspid valve regurgitation (n = 1), aortic, mitral and tricuspid valve regurgitation (n = 2) and restrictive membranous ventricular septal defect (n = 1). Diagnosis was performed using clinical examination, auscultatory findings and echocardiography. Echocardiographic anatomic and functional measurements confirmed enlargement of 1 or both left cardiac chambers in all 4 horses (Table 1 and Supporting Information Table_Supp_ 1). All horses in this cohort were regularly clinically examined for signs of pulmonary disease without any abnormal findings over the previous 12 months.

**TABLE 1:** Echocardiographic and thoracic ultrasound characteristics of the subclinical and clinical cohort

		H1_PE_		H2_PE_	
	Compensated LCVO	Before furosemide	After furosemide	Before furosemide	After furosemide
n	4	1	1	1	1
*Echocardiographic dimensions*					
Left ventricular diameter end‐diastole (LVIDd) (cm)	14.6 (13.2‐15.70)	19.13	19.7	11.7	11.4
Left ventricular diameter end‐systole (LVIDs) (cm)	9.15 (9.03‐11.0)	10.48	10.64	7.3	7.7
Left atrium long axis (cm)	14.45 (13.70‐15.6)	19.0	19.2	16.9	15.4
Aortic root diameter (cm)	8.58 (7.55‐9.15)	8.38	8.48	6.3	6.3
Aortic valve diameter (cm)	5.97 (5.74‐6.53)	7.24	7.15	5.5	5.2
Left atrium/Aorta (2D)	1.49 (1.19‐1.62)	1.72	1.67	3.09	2.97
Pulmonary root diameter (cm)	7.37 (6.56‐7.66)	9.28	9.53	7.4	6.5
Pulmonary valve diameter (cm)	5.37 (4.65‐5.83)	6.44	6.22	6.7	5.9
PA : Ao	0.86 (0.76‐0.96)	1.11	1.12	1.17	1.03
Fractional shortening (%)	37.90 (30.2‐41.1)	45.2	46	37	32
Left‐ventricular ejection fraction (%)	67.3 (54.3‐69.4)	71.5	72.3	67	66
*Pathology on ultrasound of pleural cavity and pulmonary tissue*					
Peripheral irregularity	Yes in 4 horses	Yes	Yes	Yes	Yes
Lung consolidation	Yes in 2 horses	Yes	Yes	Yes	Yes
Pleural effusion	Yes in 0 horses	Yes	Yes	Yes	Yes (less)
B‐Lines	Yes in 0 horses	Yes	Yes	Yes	Yes

*Notes*: Numerical values are reported as median (range). Pathology of the pleural cavity was evaluated over 4 sites on each hemithorax, where the presence of the characteristic in any of the 4 sites led to a positive classification (“yes”) of the horse for this characteristic.

Abbreviations: Ao, aorta; H_PE_1&2, horse with pulmonary edema 1 and 2, respectively; LCVO, left‐sided cardiac volume overload; n, number of horses; PA, pulmonary artery.

#### 2.3.2 Horses with decompensated LCVO

One Standardbred gelding (body weight 448 kg, aged 26 years) from the teaching herd with chronic mitral and aortic valve regurgitation (horse with PE 1; H1_PE_) and an Arabian stallion (body weight 480 kg, aged 8 years) admitted to the University of Zurich Equine Hospital with chronic mitral valve regurgitation and acute atrial fibrillation (horse with PE 2; H2_PE_), both with left‐sided CHF with presumed alveolar PE were included in this cohort as decompensated forms of LCVO (Supporting Information Table_Supp_ 2). Clinical diagnosis in both horses was confirmed with echocardiography and thoracic ultrasound (Table 1 and Supporting Information Table_Supp_ 2). Impairment of global gas exchange was monitored by arterial blood gas measurements. Arterial blood samples were collected anaerobically from the left facial or right carotid artery and analyzed using a regularly calibrated blood gas machines (Radiometer ABL800 FLEX, Radiometer Medical ApS, Brønshøj, Denmark). Calculation of the alveolar‐to‐arterial oxygen partial pressure difference (P_A−a_O_2_) was performed based on the formula {(FIO_2_/100) × (Atmospheric pressure − 47) − (PaCO_2_/0.8)} − {PaO_2_}, where FIO_2_ is fraction of inspired oxygen, PaO_2_ and PaCO_2_ partial pressure of arterial oxygen and carbon dioxide, respectively.

The H1_PE_ was administered furosemide once a day (Furosemide, Troy laboratories PTY. Limited, Glendenning, Australia) 1 mg/kg IV for 7 days. Clinical examination was repeated daily. Pulmonary ultrasound and EIT measurements were repeated after a 7‐day course of furosemide. In H2_PE_, furosemide 1 mg/kg IV was administered (Dimazon, MSD Santé Animale, Beaucouze, France) followed by a continuous rate infusion of 0.12 mg/kg/h starting 1 hour after the bolus. Clinical examination, pulmonary ultrasound and EIT measurements were repeated 12 hours after the administration of the furosemide bolus.

### 2.4 Data collection and analysis

#### 2.4.1 Clinical data and ultrasound examination

All horses underwent a thorough clinical examination including pulmonary and cardiac auscultation. Rebreathing bag examination was performed to assess pulmonary sounds in all horses except H1_PE_ and H2_PE_ due to clinical instability.

Ultrasound examinations of the lungs were performed using a Philips CX50 with a L12‐3 linear array transducer at a frequency of 5 MHz (Philips ultrasound, Bothell, Australia) in all horses in group LCVO but H2_PE_, in which a GE LOGIQ S8 XDclear 2.0+ ultrasound with a broad‐spectrum convex C1‐6 probe was used (GE Healthcare, Glattbrugg, Switzerland). The presence of pleural and parenchymal abnormalities was recorded in 4 areas of each hemithorax: (a and b) cranioventral and craniodorsal at 6th intercostal space (ICS); (c) at a level 10 cm proximal to the point of the shoulder within the 10th ICS; (d) at the level of the tuber coxae within the 15th ICS. Both sides of the thorax were evaluated. Thoracic ultrasound images were viewed for evidence of peripheral pulmonary irregularities, lung consolidation, pleural effusion and B‐lines over 4 sections of both hemithoraces. If there was evidence of any of the aforementioned lesions in 1 or more of the bilaterally evaluated sections, a “yes” was recorded for this lesion for this horse.

Transthoracic echocardiography was performed in a standard manner^22^, ^23^, ^24^ by the same experienced investigator (DB) using a Philips CX50 (Philips Ultrasound, Bothell, Australia) with an S5‐1 phased array transducer at a frequency of 1.7/3.6 MHz (octave harmonics) in the compensated LCVO cohort as well as in H1_PE_. Transthoracic echocardiography was performed in a standard manner in H2_PE_ using a GE Vivid 7 Ultrasound system (GE Healthcare, Glattbrugg, Switzerland) with an M4S phased array transducer operated at a frequency of 1.7/3.6 MHz (octave harmonics) by an experienced investigator. For timing purposes, a single‐lead base‐apex ECG was simultaneously recorded in all horses. Recordings were stored as cine‐loops in digital raw data format for subsequent offline analysis (EchoPAC; GE Healthcare Glattbrugg, Switzerland and Springfield Central, Australia). Two to 4 representative nonconsecutive cardiac cycles were measured and averaged for each variable. All measurements were performed retrospectively, following a predetermined measurement protocol that was used throughout data collection. The following variables were measured or calculated: Left ventricular diameter end‐diastole and end‐systole, left atrium long axis, aortic root and valve diameter, left atrium to aorta ratio (in 2‐dimensional mode), pulmonary artery root and valve diameter, pulmonary artery to aorta ratio, left ventricular fractional shortening and ejection fraction.

### 2.5 EIT measurements

After clinical and ultrasound data were recorded, the hair over the thorax directly caudal to the scapula (5th‐6th ICS) was moistened with water and ultrasound gel was applied circumferentially in this region. Thereafter, a custom‐built EIT belt with 32 electrodes was placed under slight tension around the thorax on top of the gel. The belt was positioned caudal to the inflection point of the withers, crossed the mid thorax at the level of the 6th ICS and came to lie 3‐5 cm behind the elbow. The belt was connected to the EIT device (BBvet, SenTec AG, Landquart, Switzerland). Several minutes were allowed for acclimatization. When the horse was breathing quietly, EIT data were collected over 3 minutes or until at least 10 motion artifact‐free breaths were recorded. A modified Graz consensus reconstruction algorithm for EIT (GREIT)^25^ was used to generate 47 EIT images per second for each horse, representing breathing‐related regional changes of impedance. Additional details on the use of EIT technology and image reconstruction are available.^8^, ^25^, ^26^, ^27^ At the completion of data acquisition, the belt was removed, the gel hosed down with water and the horse returned to the paddock or stable.

A sequence of at least 6 and a maximum of 10 consecutive stable, artifact‐free breaths was retrospectively selected for each horse, depending on the respiratory rate and cooperation of the horse during the measurement period, using dedicated software (ibeX, SenTec, Landquart, Switzerland).^28^ Of the selected breaths, conventional EIT variables were exported to a spreadsheet (Excel for MAC, Microsoft Cooperation, Redmond, Washington). In addition to these EIT variables, EIT raw data were exported and analyzed using custom software developed by the authors for the generation of nonconventional EIT variables. The EIT lung regions of interest (ROIs) were predetermined by a finite element model based on horse anatomy.^29^, ^30^ Briefly, the finite element model was created based on a transverse postmortem section of the thorax obtained from a frozen equine specimen at the level of the 6th ICS.^30^ These sections were used to map functional EIT images onto the relevant ROIs within the entire thoracic image.

#### 2.5.1 Conventional EIT variables

**FIGURE 1:**
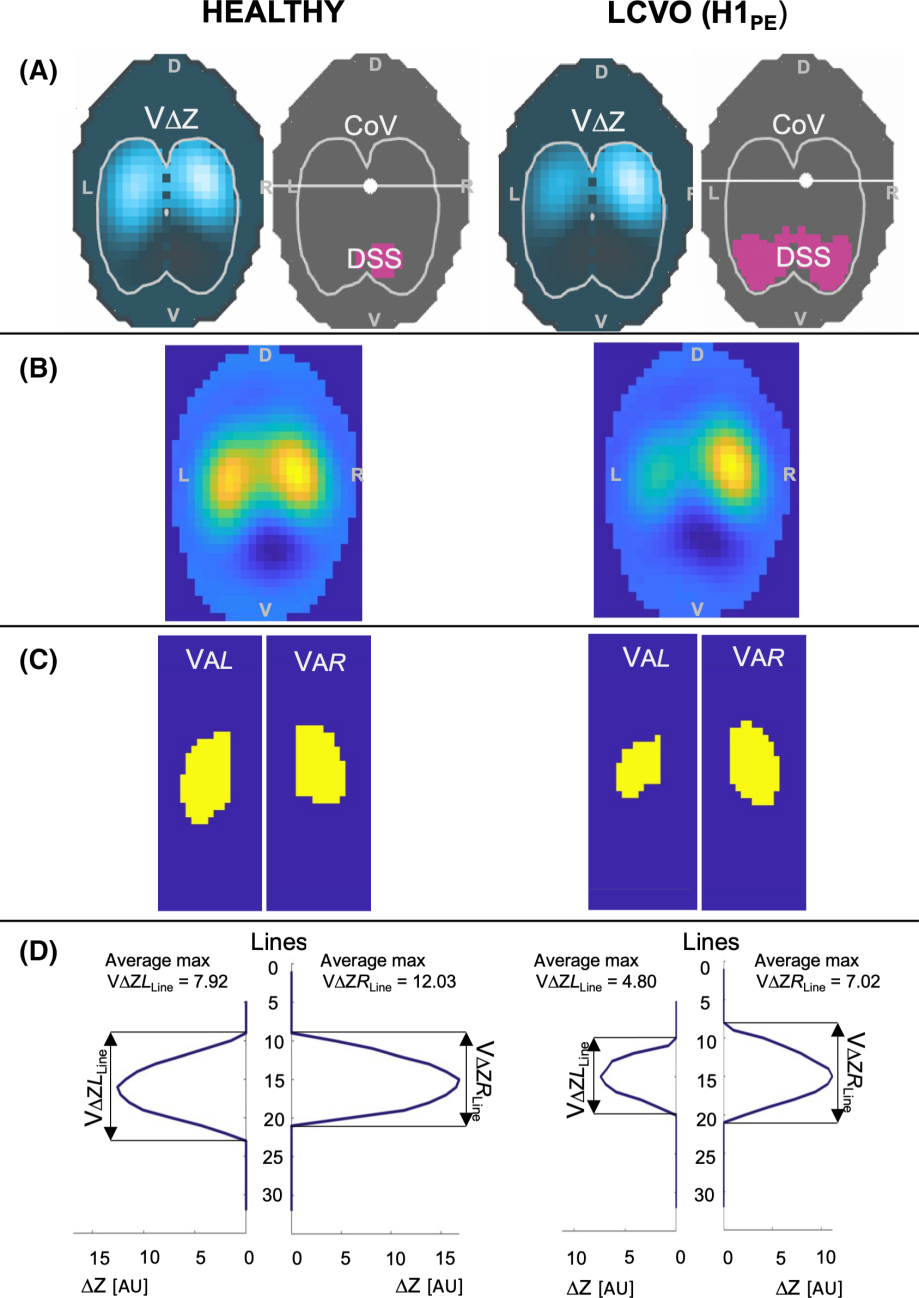
Electrical impedance tomography (EIT) images and graphical illustration of variable calculations from 1 horse in the control group (healthy) and 1 horse with decompensated left‐sided cardiac volume overload (LCVO‐H1_PE_). A, EIT images showing ventilation‐induced impedance changes (VΔZ) in blue within the predefined lung regions (white outline). The dark gray image shows the position of the center of ventilation (CoV) as white dot and the dependent silent space (DSS) as pink area within the same predefined lung region. B, EIT image for each horse reconstructed from the same raw data as (A) without predefined lung regions. C, Illustration of the area of detectable impedance change in (B) representing the ventilated area of the left (VA*L*) and right (VA*R*) lung field. D, Pixel line distribution over 32 horizontal lines for the left and the right lung showing the maximal V∆Z for each line; from this graph the number of lines with detected impedance change can be evaluated for the left (VΔZ*L*_Line_) and right (VΔZ*R*_Line_) lung field. The value of maximal V∆Z for each line is used to calculate the average maximum impedance change for each lung (average max VΔZ*L*_Line_ and VΔZ*R*_Line_)

1. The ventral to dorsal (CoV_VD_) and right to left (CoV_RL_) centers of ventilation express the geometric focal point of overall ventilation as a single value. The vertical (CoV_VD_) or horizontal (CoV_RL_) position of the CoV is expressed as a percentage of the ventro‐dorsal or right‐left extension of the lung region, respectively. For CoV_VD_ and CoV_RL_ 0% refers to ventilation occurring in the most ventral part of the lung and the right lung respectively, whereas 100% refers to that in the most dorsal part and the left lung, respectively (Figure 1A).^31^
2. Ventilation‐induced impedance changes within the lung field below 10% of the maximum value were determined and these regions defined as “silent space.” They were divided into dependent (DSS) or nondependent silent space (NSS) and expressed as a percentage of the entire lung region (Figure 1A).^32^
3. The global impedance change of all pixels between the beginning and end of inspiration within the lung field was calculated for each breath and used as a surrogate for tidal volume, expressed by EIT (VΔZ; Figure 1A).^13^
4. To describe the ventro‐dorsal distribution of ventilation during inspiration regionally on a more detailed pixular level, the entire lung field was divided by 4 horizontal lines into 4 vertical portions of equal height (ventral 25% = VΔZ_*V*_; central ventral 25% = VΔZ_*CV*_; central dorsal 25% = VΔZ_*CD*_; dorsal 25% = VΔZ_*D*_).

#### 2.5.2 Nonconventional EIT variables

(1) Variables based on pixel line distribution:

In this study, impedance change was evaluated at the pixular level of the 32 horizontal lines. The ventilated pixel lines were assessed to gain a better understanding of the dorso‐ventral distribution of ventilation in horses with LCVO in the left and right lung (Figure 1D):

- Number of ventilated lines: all 32 horizontal matrix lines were assessed for each breath. The number of lines with detected impedance change was recorded for the right (VΔZ*R*_Line_) and the left (VΔZ*L*_Line_) lung field separately.
- Average maximum impedance change: Maximum impedance change was identified for each of the horizontal matrix lines. The average of these maximums was calculated for each lung (right: avg‐max VΔZ*R*_Line_; left: avg‐max VΔZ*L*_Line_).
- Graphical illustration of impedance change over 32 matrix lines.

(2) Ventilated area (Figure 1C): The percentage of pixels of the total image representing the ventilated area of the right (VA*R*) and left (VA*L*) lung field identified by detectable impedance change on an individual pixular level.

(3) Global inhomogeneity index (VGI): The differences in impedance variation between each pixel and the median value of all pixels are calculated and normalized to the sum of impedance values using a previously described formula.^6^, ^33^, ^34^ The overall heterogeneity of tidal volume distribution was described as an average over 4 breaths, whereby a smaller VGI represents a more homogeneous distribution, and a larger VGI indicates a more inhomogeneous ventilation associated with altered lung function.

(4) Peak inspiratory and expiratory flow: flow variables were analyzed using EIDORS^35^ and author‐written software using GNU octave. Global peak inspiratory (PIF_EIT_) and expiratory (PEF_EIT_) gas flows were calculated as the first derivative of the global impedance change. The flow variables for inspiratory (PIF_EIT_/VΔZ) and expiratory (PEF_EIT_/VΔZ) flow were subsequently normalized to the EIT derived tidal volume VΔZ.

### 2.6 Statistics

Normally distributed data are summarized as mean ± SD. Results are summarized as median (range) for nonnormal data. As no research was available on which to base the standard deviations of any EIT outcome variable in horses with cardiac disease, no a priori power analysis was performed for this study.

Normality was assessed using a Shapiro‐Wilk test and visualization of Q‐Q plots. Normal data for horses with LCVO were compared to the healthy cohort using an unpaired *t*‐test against a 2‐sided *P* value <.05, using the Pooled or Satterthwaite correction depending on whether the assumption of equal variance was met or not, respectively. Nonnormal data was compared using a Mann‐Whitney sum test against a 2‐sided *P* < .05 using the Z approximation. For normal data, the mean difference and its 95% confidence interval is reported. For nonnormal data, the difference in the median ranks is reported. Analysis was performed using SAS 9.4 (SAS institute, Cary NC) and *P* ≤ .05 was considered statistically significant. Observations made across the summary statistics for compensated and decompensated LCVO horses, as well as before and after treatment in H1_PE_ and H2_PE_, are reported.

## 3 RESULTS

### 3.1 Comparison healthy and LCVO horses

The 8 healthy horses included 5 geldings and 3 mares (6 Standardbreds, 2 Thoroughbreds) with a mean (±SD) age of 10.5 (±2.8) years and weight of 491.1 (±77.1) kg. Mean heart rate, respiratory rate and rectal temperature were 39.25 (±6.23) beats per minute (bpm), 7 (±3.04) breaths per minute (brpm) and 37.31 (±0.27)°C, respectively.

The 6 LCVO horses included 5 geldings and 1 stallion (1 Standardbred cross, 2 Thoroughbreds, 1 Standardbred, 1 Arabian horse) with a mean (±SD) age of 18.0 (±11.5) years and bodyweight of 532.0 (±40.9) kg. Mean heart rate, respiratory rate and rectal temperature were 48 (±2.83) bpm, 19.60 (±2.19) brpm and 37.9 (±0.2)°C, respectively. Heart rate (*P* = .006) and respiratory rate (*P* < .001) were significantly higher in horses with LCVO compared to healthy. Thoracic ultrasound of all LCVO horses showed peripheral irregularities and all but 2 also had ultrasonographic evidence of lung consolidation. Pleural effusion and B‐lines were evident in H1_PE_ and H2_PE_ (Table 1).

**FIGURE 2:**
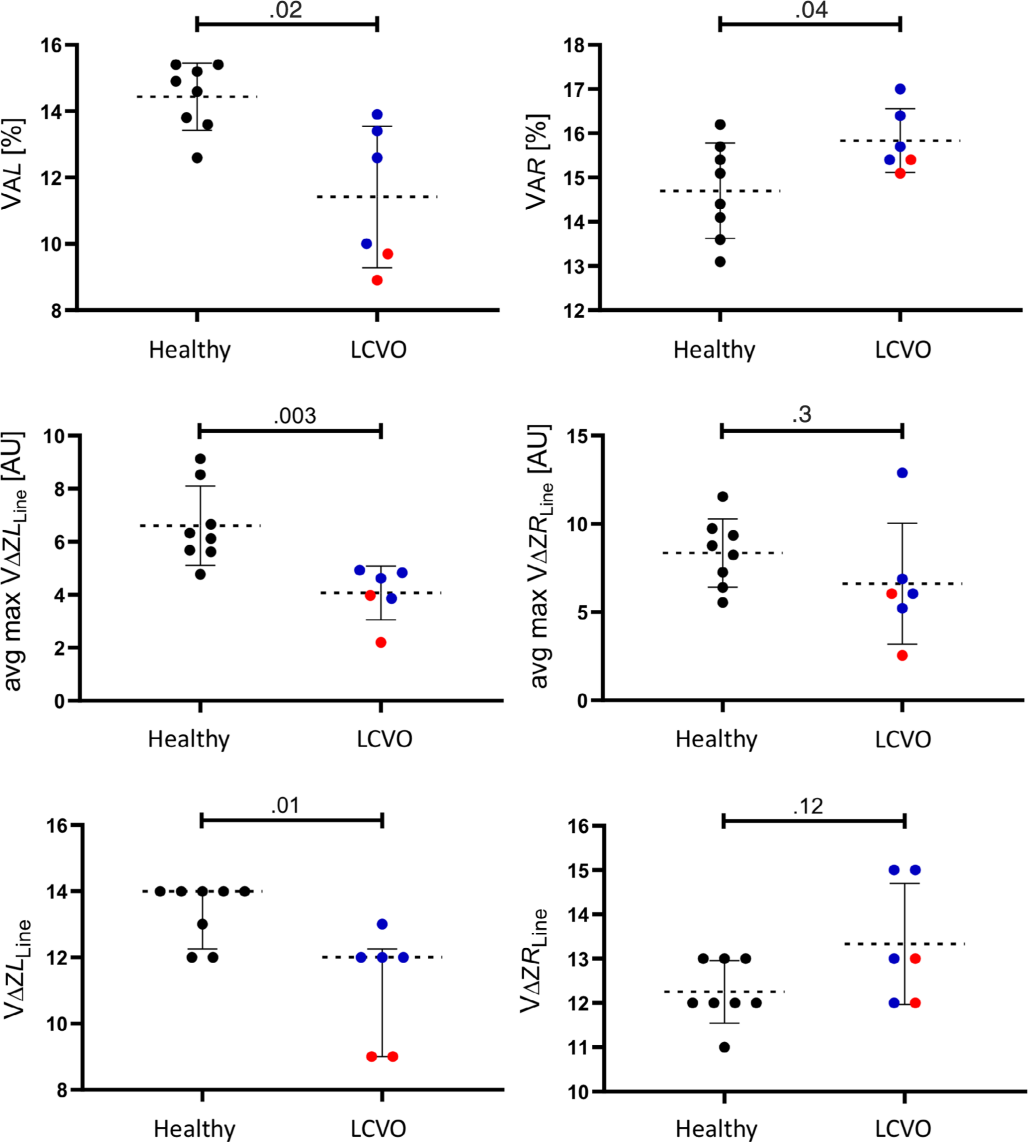
Scatter plot graphs of nonconventional electrical impedance tomography (EIT) variables evaluated from healthy horses (n = 8, black dots) and horses with compensated (blue dots) and decompensated (red dots) left‐sided cardiac volume overload (LCVO; n = 6) describing the ventilated area over the right (VA*R*) and left (VA*L*) lung field, average maximum impedance change (right: avg‐max VΔZ*R*_Line_; left: avg‐max VΔZ*L*_Line_) and amount of ventilated matrix lines (VΔZ*R*_Line_; VΔZ*L*_Line_)

**FIGURE 3:**
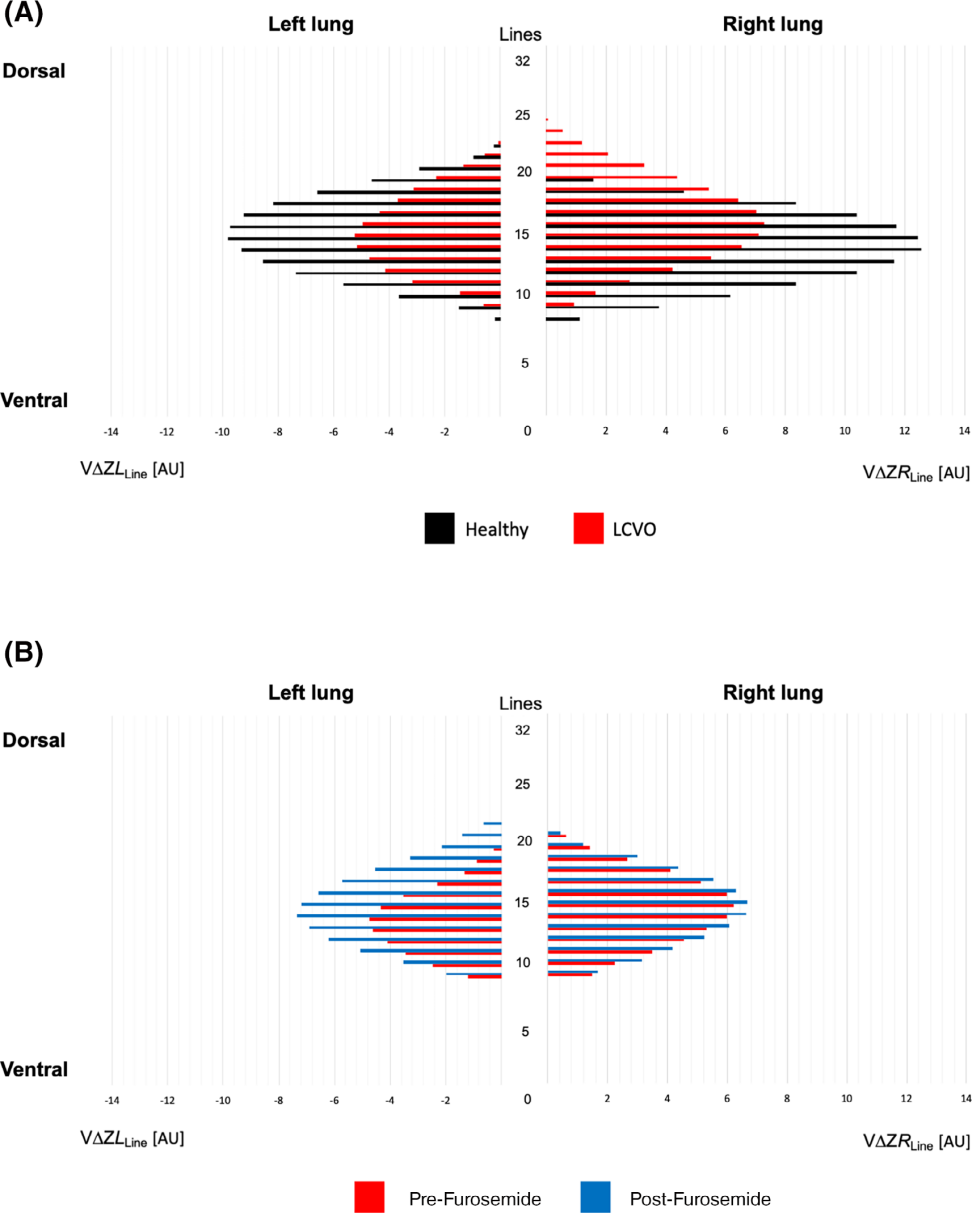
Graphical linear‐plane distribution of the left and right lungs of standing horses, displaying the 32 matrix lines of the electrical impedance tomography (EIT) image on the y‐axis and the mean of total impedance change per line (VΔZ_Line_) of all horses per group on the x‐axis in arbitrary units (AU). A, Healthy horses (Healthy; n = 8) and horses with left sided cardiac volume overload (LCVO; n = 6). B, Horses with decompensated LCVO before furosemide administration (Pre‐Furosemide; n = 2), and after furosemide administration (Post‐Furosemide; n = 2)

Most EIT variables were normally distributed, except for CoV_RL_, NSS and ΔZ*L*_Line_ (Table 2). No conventional EIT variable was significantly different between cohorts. For LCVO horses, VA*L* (mean difference 3.02; 95% confidence interval [CI] of mean difference .77‐5.2; *P* = .02) was significantly smaller, and avg‐max ΔZ_avgL_ (mean difference 2.54; 95% CI 1.07‐4.00; *P* = .003) and ΔZ_LineL_ (median difference 5.40; 95% CI of median difference 1.71‐9.09; *P* = .01) were significantly lower, whereas VA*R* (mean difference − 1.13; 95% CI: −2.18 to −.08; *P* = .04) was significantly larger compared to healthy horses (Figures 2 and 3). There were no significant differences between cohorts for other nonconventional EIT variables. Graphical linear‐plane distribution of ventilation is shown in Figure 3.

**TABLE 2:** Electrical impedance tomography variables in a healthy cohort and horses with compensated/decompensated cardiac disease

	Healthy	LCVO					
		Compensated + decompensated	Compensated	H1_PE_		H2_PE_	
				Before furosemide	After furosemide	Before furosemide	After furosemide
n	8	6	4	1	1	1	1
*Conventional EIT variables*							
V**Δ**Z (AU)	3.47 ± 0.54	2.86 ± 1.03	3.34 ± 0.67	2.65	2.58	1.13	1.87
CoV_RL_ (%)	50.14 (48.76‐51.80)	47.69 (44.06‐56.49)	46.39 (45.25‐49.25)	44.06	45.77	56.49	56.25
CoV_VD_ (%)	66.87 ± 4.42	63.47 ± 6.54	59.43 ± 2.36	72.23	70.79	70.91	65.25
NSS (%)	0	0.22 (0.0‐0.76)	0.33 (0–0.76)	0	0	0	0
DSS (%)	7.29 ± 0.37	8.51 ± 3.33	6.98 ± 2.67	9.85	9.24	13.30	9.05
V**Δ**Z_*V*_ (AU)	1.12 ± 0.9	2.46 ± 2.05	3.53 ± 1.57	0.10	0.01	0.57	1.06
V**Δ**Z_*CV*_ (AU)	14.44 ± 5.21	20.61 ± 10.50	27.21 ± 3.05	7.70	8.71	7.13	17.95
V**Δ**Z_*CD*_ (AU)	41.84 ± 2.40	43.21 ± 2.24	43.88 ± 1.85	39.71	42.29	44.02	45.16
V**Δ**Z_*D*_ (AU)	41.63 ± 7.73	33.72 ± 13.42	25.38 ± 4.35	52.50	48.99	48.29	35.83
*Nonconventional EIT variables*							
VA*R* (%)	14.70 ± 1.08*	15.83 ± 0.72*	16.13 ± 0.72	15.40	15.60	15.10	15.70
VA*L* (%)	14.44 ± 1.01*	11.42 ± 2.13*	12.48 ± 1.73	8.90	13.10	9.70	12.50
V**Δ**Z*R*_Line_	12.3 (11‐13)	13.3 (12–15)	13.8 (12–15)	13	13	12	13
V**Δ**Z*L*_Line_	13.4 (12‐14)*	11.2 (9–13)*	12.3 (12‐13)	9	11	9	11
Avg‐max∆Z_avgR_ (AU)	8.35 ± 1.93	6.61 ± 3.42	7.76 ± 3.49	6.04	5.75	2.55	3.76
Avg‐max∆Z_avgL_ (AU)	6.61 ± 1.49*	4.07 ± 1.02*	4.56 ± 0.48	3.97	6.92	2.2	3.09
VGI	0.43 ± 0.03	0.42 ± 0.01	0.42 ± 0.02	0.407	0.460	0.409	0.418
PIF_EIT_ (AU)	1.98 ± 0.25	1.78 ± 0.41	1.99 ± 0.19	1.68	1.51	1.04	1.63
PEF_EIT_ (AU)	2.35 ± 0.62	2.08 ± 0.49	2.20 ± 0.13	2.52	2.59	1.13	1.84
PIF_EIT_/V**Δ**Z	0.58 ± 0.08	0.67 ± 0.18	0.62 ± 0.17	0.63	0.59	0.92	0.87
PEF_EIT_/V**Δ**Z	0.68 ± 0.16	0.78 ± 0.19	0.68 ± 0.14	0.947	1.004	0.999	0.985

*Notes*: Numerical values for cohorts are reported as mean ± SD or median (range). Significant differences between healthy and LCVO cohorts are indicated with *.

Abbreviations: avg‐max∆Z_avgR_ and avg‐max∆Z_avgL_, average maximum impedance change for each horizontal matrix line for the right and the left lung, respectively; CoV_RL_, right to left center of ventilation; CoV_VD_, ventral to dorsal center of ventilation; DSS, dependent silent space; EIT, Electrical impedance tomography; H_PE_1&2, horse with pulmonary edema 1 and 2, respectively; LCVO, left‐sided cardiac volume overload; n, number of horses; NSS, nondependent silent space; PEF_EIT_, global peak expiratory gas flow; PEF_EIT_/VΔZ, peak expiratory gas flow normalized by tidal volume; PIF_EIT_, global peak inspiratory gas flow; PIF_EIT_/VΔZ, peak inspiratory gas flow normalized by tidal volume; ROI, region of interest ROI divided by 4 horizontal lines into 4 stacked vertical portions (ventral = VΔZ_V_; central ventral = VΔZ_CV_; central dorsal = VΔZ_CD_; dorsal = VΔZ_D_); VA*R* and VA*L*, ventilated area of the right (VA*R*) and left (VA*L*) lung; VGI, global inhomogeneity index; VΔZ (AU), tidal volume in EIT arbitrary units; VΔZ*R*_Line_ and VΔZ*L*_Line_, number of ventilated lines over 32 horizontal matrix lines for the right (VΔZ*R*_Line_) and the left (VΔZ*L*_Line_) lung.

### 3.2 Observations on compensated and decompensated LCVO horses, and the effect of furosemide administration in decompensated LCVO horses

Horses with compensated LCVO did not show any abnormalities during the clinical examination. Tachycardia (defined as heart rate > 48 bpm) and clinical signs of respiratory disease were found in H1_PE_ and H2_PE_ (Supporting Information Table_Supp_ 2). Both horses with decompensated LCVO had pulmonary roots larger than aortic roots, supportive of pulmonary hypertension (Table 1). Further echocardiographic markers of pulmonary hypertension (such as tricuspid regurgitation velocity) were not performed.

**FIGURE 4:**
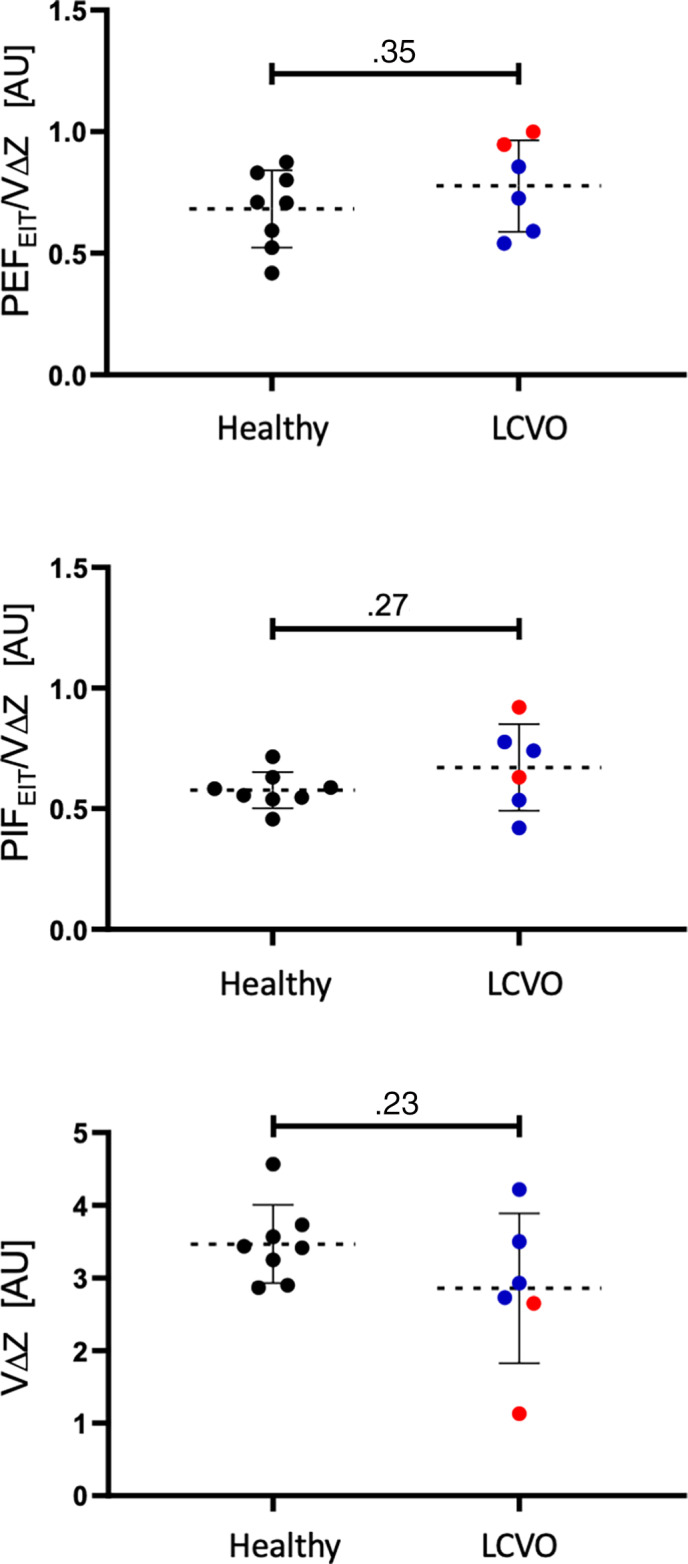
Scatter plot graphs of peak inspiratory (PIF_EIT_) and expiratory flow (PEF_EIT_) normalized by total impedance change (VΔZ) and VΔZ as surrogate for tidal volume measured by electrical impedance tomography evaluated from healthy horses (n = 8, black dots) and horses with compensated (blue dots) and decompensated (red dots) left‐sided cardiac volume overload (LCVO; n = 6)

Based on observation of the means, VΔZ, PIF_EIT_, VA*L*, VΔZ*L*_Line_ were lower, and CoV_VD_, DSS, and PEF_EIT_/VΔZ were estimated as higher in the horses with decompensated LCVO than in compensated LCVO (Figures 2 and 4). Furthermore, the ventilation was observed to be estimated lower in the ventral and central‐ventral lung regions and higher in the most dorsal lung regions in the horses with clinical signs of alveolar PE than in the horses with compensated LCVO.

Further observation of variables for the H1_PE_ and H2_PE_ horses showed that clinical measurements and echocardiography findings improved in response to administration of furosemide (Tables 1 and 2). An improvement toward the values observed in healthy and compensated LCVO horses was seen for VΔZ (18%), DSS (21%), VA*L* (38%), VΔZ*L*_Line_ (22%), and avg‐maxVΔZ*L*_Line_ (62%) after furosemide. An increased in VΔZ_*CV*_ and VΔZ_*CD*_, but decrease in VΔZ_*D*_ was observed after administration of furosemide in both horses.

## 4 DISCUSSION

This study has shown that EIT measurements can be used to detect differences in the distribution of ventilation in horses with LCVO compared to normal horses and are consistent with less ventilation in the left lung with an increased ventilated area of the right lung. This decrease in left‐sided ventilation and a concurrent increase in ventilation of the dorsal parts of the lungs was most obvious in 2 horses with decompensated disease with alveolar PE. Both alterations in ventilation progressed in response to treatment, toward the measurements taken in healthy horses. These results confirm our hypotheses, that EIT‐derived variables would be different between healthy horses and horses with LCVO of different degrees. Our observations also support an expectation that these differences become less after diuretic treatment in decompensated LCVO horses.

Cardiogenic pulmonary changes can be difficult to diagnose in horses with conventional diagnostic approaches used in other species. Clinical signs of primarily interstitial accumulation of extravascular lung water and tissue fibrosis due to high pulmonary pressure in early or mild heart failure might be limited to increased respiratory rate and crackles or moist bronchovesicular sounds.^36^ In this study, horses with compensated LCVO did not demonstrate adventitious lung sounds on thoracic auscultation. Only once advanced will alveolar PE cause dyspnea, coughing and profuse frothy nasal discharge, such as seen in H2_PE_. Beside clinical signs, thoracic ultrasound can be used to visualize peripheral pleural and parenchymal lesions. Several abnormalities were observed in our horses with LCVO with at least 1 abnormality in every horse confirming the presence of lung changes in this cohort (Table 1). However, with regards to extravascular lung water accumulation, changes are most pronounced at the central hilar area, and could be missed by diagnostic ultrasonography as well as radiology in large animals, especially when interstitial or a low quantity of fluid accumulation in the alveolar space is present.^36^ Electrical impedance tomography has been shown to be able to detect cardiogenic accumulation of extravascular lung water in humans, pigs and dogs, mainly by revealing shifts in the distribution of ventilation.^14^, ^15^, ^16^, ^17^, ^18^, ^19^

**FIGURE 5:**
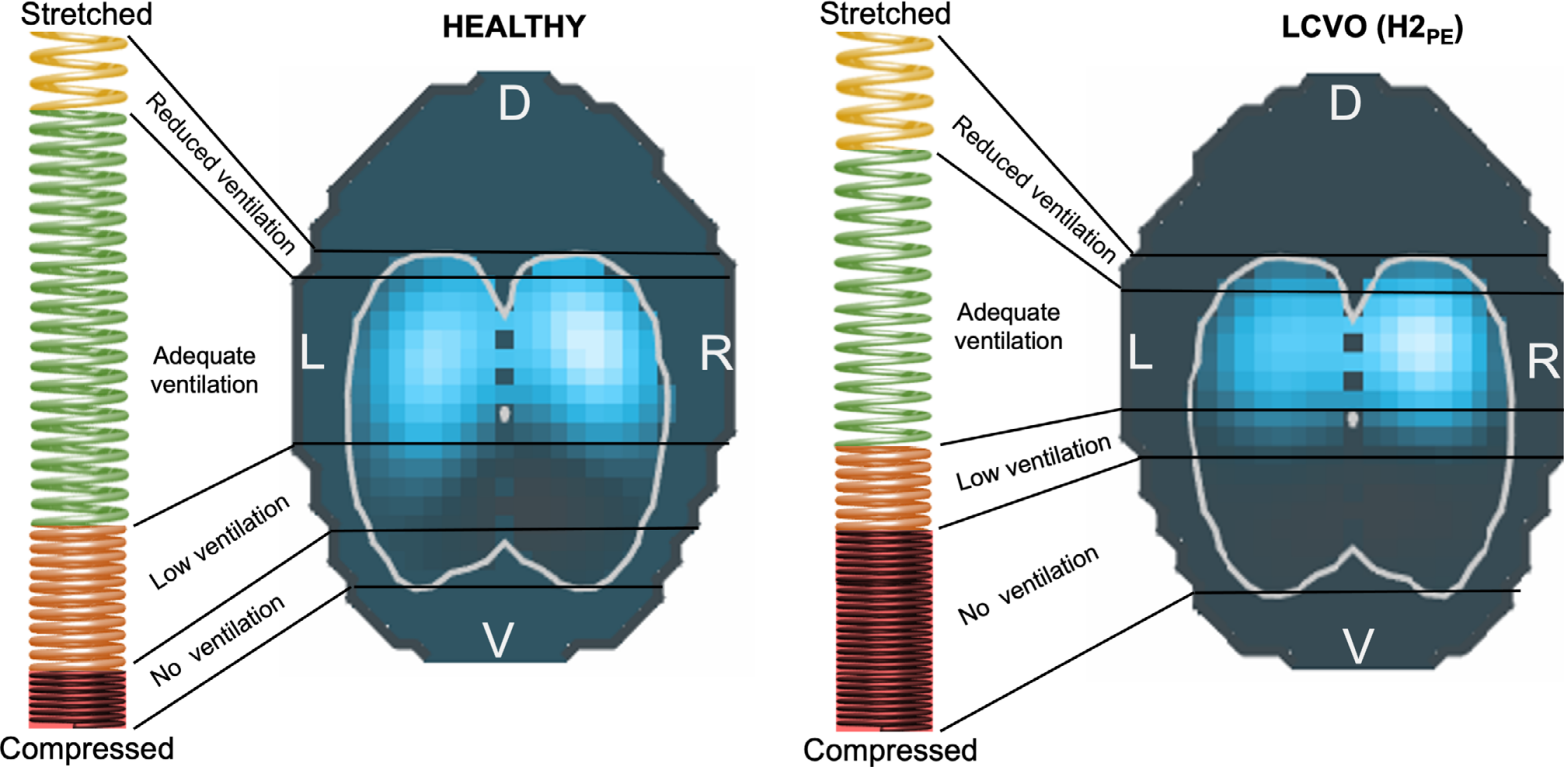
The “spring model” illustrating the slinky effect of gravity on the distribution of ventilation. The differences in the coil stretch of the spring and the EIT image between a healthy horse (left) and a horse with left‐sided cardiac volume overload (LCVO; right) are displayed. The vertically oriented spring is stretched dorsally and compressed at the bottom. The corresponding EIT image demonstrates the areas of detected impedance change in light blue. An increase in extravascular lung water (right) leads to a greater number of coils in the dependent portion of the spring, which is analogous to a greater density of the lung tissue and therefore less ventilation detected in the EIT image

To inspect the differences in the distribution of ventilation we initially created graphs showing the impedance change for each of the 32 horizontal matrix lines of the EIT functional image for healthy and LCVO horses plus the decompensated horses before and after administration of furosemide (Figure 3). Additionally, the EIT images generated by the analyzing software were visually inspected. Figures 1 and 5 demonstrate comparisons of the generated EIT images from healthy and horses with LCVO. These illustrations confirmed the hypothesized difference in the distribution of ventilation between cohorts, but also indicated that conventional EIT variables will unlikely be able to verify the alterations in horses with LCVO. Based on these initial observations, nonconventional EIT variables describing the ventilated area and line distribution were constructed to demonstrate significant numerical differences between cohorts, which were present for some of the novel EIT variables.

The observation that the left lung was more affected by LCVO was unexpected. In people, 1‐sided PE of the right lung has been reported as a complication of acute mitral valvular insufficiency.^37^ This is explained by the vector of the retrograde blood flow across the mitral valve that is directed into 1 of the right superior pulmonary veins in humans. In horses, the anatomy of the pulmonary veins differs^38^ and reversal flow might favor venous congestion and edema location in the left lung. Another reason for a predominantly left‐sided decrease in ventilation in LCVO horses might be the position of the heart, sitting cranio‐ventrally and slightly left‐sided within the thorax. In humans with severe congenital heart disease and altered cardiac dimensions, bronchial compression has been described.^39^, ^40^ The enlarged left atrium of horses with LCVO might have a direct compressive effect on the left mainstem bronchus, restricting the airway lumen and redirecting the gas toward the right lung, and could explain the increased ventilated area of the right lung. Such a change in gas flow, however, would be expected to cause inhomogeneity in inflation of the right and left lung units, as a narrowing in airway diameter would alter the velocity of the gas within the airways following Bernoulli's principle. This was not confirmed, as the global inhomogeneity index calculated from EIT measurements between healthy and diseased horses was similar, making unilateral airway compression less likely.

The physical explanation for changes in the distribution of ventilation in LCVO is the increase in extravascular lung water in conjunction with gravity as best visualized by a slinky spring fixed at the top and the bottom—the “slinky effect” (Figure 5).^41^, ^42^, ^43^ When LCVO leads to rising intrapulmonary hydrostatic pressures, fluid is forced from the pulmonary capillaries into the dependent pulmonary interstitium and free water accumulates in the alveoli. This causes the alveolar matrix to expand dorsally, thus leading to a dorsal shift in ventilation distribution. In addition, the matrix collapses in the ventral areas, decreasing the overall ventilated area^18^ (Figure 5, LCVO). Conventional EIT variables to describe such a shift include the 4 stacked ROIs from ventral to dorsal, CoV_VD_, and the Silent Spaces.^31^, ^32^, ^43^ A statistically significant difference was not detected in these conventional variables between the cohorts. When assessing only the horses with decompensated LCVO, however, changes in accordance with the proposed slinky effect were observed. In these 2 horses, less ventilation was detected in the ventral and central‐ventral ROIs and more in the most dorsal ROI when compared to horses with compensated LCVO (Table 2). Similarly, CoV_VD_ was more dorsal and DSS higher in the decompensated LCVO horses. In addition, changes in these variables demonstrating a ventral shift of ventilation distribution were observed after administration of furosemide to the 2 horses with decompensated LCVO.

It is worth mentioning that based on the standard diagnostic measurement of P_a_O_2_ and the calculation of P_A‐a_O_2_ to evaluate the severity of alveolar PE both horses with clinical signs showed an elevated P_A‐a_O_2_ value that decreased in response to furosemide administered IV, indicating a reduction in venous admixture. However, both horses, although showing obvious signs of pulmonary disease and PE were not hypoxemic at any time point. Therefore, global markers of altered ventilation distribution might be less accurate in evaluating cardiogenic lung disease in comparison with modalities that can detect regional ventilation differences.

## 5 LIMITATIONS

The study is limited to a small number of horses and variability in stages of cardiac disease. Despite our small sample size, we were able to detect significant differences in novel EIT variables. Thus, the results of our study provide motivation to pursue further investigations of EIT to describe pulmonary changes as a sequela of cardiac disease and could direct future, larger scale studies.

In this study, vital variables, thoracic auscultation, and ultrasound were used as inclusion criteria in the LCVO cohort. No radiography was performed despite reports of relevant findings in horses with PE such as prominent pulmonary vascular markings, increased interstitial lung density, and peri‐bronchial fluid collection.^1^ While this might have given additional information for grading of the extent of pulmonary changes, there was no intent to associate magnitude of changes in EIT variables with extent of disease.

## CONFLICT OF INTEREST DECLARATION

Authors declare no conflict of interest.

## OFF‐LABEL ANTIMICROBIAL DECLARATION

Authors declare no off‐label use of antimicrobials.

## INSTITUTIONAL ANIMAL CARE AND USE COMMITTEE (IACUC) OR OTHER APPROVAL DECLARATION

Approved by the Animal Ethics Committee Murdoch University School of Veterinary Medicine (MUSVM), permit number R3205/19.

## HUMAN ETHICS APPROVAL DECLARATION

Authors declare human ethics approval was not needed for this study.

## Supporting information

**Table S1**. Clinical variables in 2 horses suffering from pulmonary edema, before and after furosemide treatment
**Table S2**. Reference values for cardiac dimensions in healthy Standardbred, Thoroughbred, and Arabian horses used for cohort allocation in this study (Patteson et al, 1995; Sleeper et al, 2014; Zucca et al, 2008)
