# Supplementary material for: Electrical impedance tomography to measure lung ventilation distribution in healthy horses and horses with left‐sided cardiac volume overload
Source: J Vet Intern Med. 2021 Aug 4;35(5):2511–23. doi: 10.1111/jvim.16227 (PMC8478054; doi:10.1111/jvim.16227)
Supplement: Supplementary file 1 — Table S1. Clinical variables in 2 horses suffering from pulmonary edema, before and after furosemide treatment Table S2. Reference values for cardiac dimensions in healthy Standardbred, Thoroughbred, and Arabian horses used for cohort allocation in this study (Patteson et al, 1995; Sleeper et al, 2014; Zucca et al, 2008) [file JVIM-35-2511-s001.docx]

**TABLE_SUPP_ 1** Reference values for cardiac dimensions in healthy Standardbred, Thoroughbred and Arabian horses used for cohort allocation in this study (Patteson et al.1995, Sleeper et al 2014, Zucca et al, 2008).

|  | **Standardbred**  (mean ± SD) | **Thoroughbred**  (mean ± SD) | **Non-elite Arabian horses**  Mean (range) |
| --- | --- | --- | --- |
| Interventricular septum (end-diastole) (cm) | 3.1 ± 0.41 | 2.85 ± 0.28 | 2.7 (2.3-3.4) |
| Interventricular septum (end-systole) (cm) | 4.48 ± 0.36 | 4.21 ± 0.46 | 4.2 (3.3-5.1) |
| Left ventricular ID (end-diastole) (cm) | 11.64 ± 1.29 | 11.92 ± 0.76 | 10.7 (9.6-12.1) |
| Left ventricular ID (end-systole) (cm) | 7.42 ± 1.05 | 7.45 ± 0.62 | 6.7 (6.0-7.8) |
| Left ventricular wall (end-diastole) (cm) | 2.55 ± 0.36 | 2.32 ± 0.38 | 2.1 (1.8-2.5) |
| Left ventricular wall (end-systole) (cm) | 3.64 ± 0.52 | 3.85 ± 0.41 | 4.0 (3.3-4.9) |
| Fractional shortening (%) | 36.2 ± 3.9 | 37.4 ± 3.9 | 38 (33-44) |
| Aortic root diameter (cm) | 7.79 ± 0.46 | 9.0 ± 0.5 | 8.4 (7.4–9.6) |
| Left atrium long axis (cm) | 11.49 ± 0.5 | 12.82 ± 0.78 | 11.9 (10.9-13.8) |
| Pulmonary artery root diameter (cm) | 5.41 ± 0.38 | 6.1 ± 0.49 | 6.3 (5.5-6.8) |
| Left Atrium/Aorta | <1.50 | <1.50 | <1.50 |

**TABLE_SUPP_ 2**  Clinical variables in two horses suffering from pulmonary edema, before and after furosemide treatment

|  | H1_PE_ | | H2_PE_ | |
| --- | --- | --- | --- | --- |
|  | **Before furosemide**  **(d0)** | **After furosemide**  **(d7 after furosemide)** | **Before furosemide**  **(d0)** | **After furosemide**  **(d1 - 12h after furosemide)** |
| Heart rate (bpm) | 56 | 52 | 125 (AFib) | 110 (AFib) |
| Respiratory rate (brpm) | 22 | 16 | 24 | 24 |
| Respiratory effort at rest | increased | normal | Increased | Increased |
| Rectal temperature (°C) | 37.0 | 36.9 | 38.1 | 37.8 |
| Cough at rest | yes | yes | Yes | Yes |
| Nasal discharge | none | none | frothy | none |
| Demeanor | quiet | normal | apathy | apathy |
| Cardiac auscultation | Systolic left sided murmur 4/6,  Diastolic left sided murmur 4/6 | Systolic left sided murmur 4/6,  Diastolic left sided murmur 4/6 | Holosystolic left sided murmur 4/6,  Holosystolic right sided murmur 3/6 | Holosystolic left sided murmur 4/6,  Holosystolic right sided murmur 3/6 |
| Thoracic auscultation | Bilateral moderate crackles cranio-ventral lung fields | Bilateral mild crackles cranio-ventral lung fields | Bilateral moderate crackles cranio-ventral lung fields | Bilateral mild crackles cranio-ventral lung fields |
| PaO_2_ (mmHg) | 93 | 99 | 86 | 89 |
| PaCO_2_ (mmHg) | 39 | 41 | 45 | 44 |
| Lactate (mmol/L) | .8 | .7 | .8 | .8 |
| P_A-a_O_2_ (mmHg) | 8 | 1 | 9 | 6 |

Abbreviations: H_PE_1&2; Horse with pulmonary edema 1 and 2, respectively; AFib, atrial fibrillation; bpm, beats per minute; brpm, breath per minute; PaO_2_, partial pressure of arterial oxygen; PaCO_2_, partial pressure of arterial carbon dioxide; P_A-a_O_2_, alveolar-to-arterial oxygen partial pressure difference; d0, measurement day 0 (before treatment); d7, measurement on day 7 after start of treatment; d0 + 12h, measurement 12 hours after start of treatment.
